# Health literacy as a moderator of menu labeling effects on fast-food ordering: an online randomized controlled trial

**DOI:** 10.1093/abm/kaag049

**Published:** 2026-08-24

**Authors:** Yiqing Skylar Yu, Alyssa Leib, Laura L Bellows, Christopher Berry, Dan J Graham, Danielle M Krobath, Megan P Mueller

**Affiliations:** Department of Psychology, Colorado State University, Fort Collins, CO 80523-1876, United States; Department of Food Science and Human Nutrition, Colorado State University, Fort Collins, CO 80523-1571, United States; Division of Nutritional Sciences, Cornell University, Ithaca, NY 14853, United States; Department of Marketing, Colorado State University, Fort Collins, CO 80523-1278, United States; Department of Psychology, Colorado State University, Fort Collins, CO 80523-1876, United States; Colorado School of Public Health, Colorado State University, Fort Collins, CO 80523-1612, United States; Department of Epidemiology and Biostatistics, Arnold School of Public Health, University of South Carolina, Columbia, SC 29208, United States; Department of Food Science and Human Nutrition, Colorado State University, Fort Collins, CO 80523-1571, United States

**Keywords:** health literacy, food labeling, fast foods, food choice behavior, sodium, dietary, randomized controlled trial

## Abstract

**Objectives:**

To examine whether functional health literacy moderates the effects of menu labeling formats on fast-food ordering behavior.

**Methods:**

A total of 2829 US adults completed an online randomized controlled survey in 2022. Participants viewed 1 of 8 menus in a 2 × 2 × 2 factorial design varying calorie labels (present/absent), sodium stoplight icons (present/absent), and “all-natural” claims (present/absent), then selected an entrée, side, beverage, and optional dessert. Linear regression models tested main and interaction effects of labeling formats and functional health literacy on total calories and sodium ordered, adjusting for sociodemographic characteristics, fast-food consumption frequency, and typical ordering patterns.

**Results:**

In covariate-adjusted within-literacy comparisons, several labeling experimental conditions were associated with lower-calorie and lower-sodium selections among participants with moderate functional health literacy, whereas no significant differences were observed among participants with low or high literacy. Significant differences among moderate-literacy participants ranged from 90 to 135 fewer calories and 123 to 154 mg less sodium compared with the control condition (nominal *P* < .05).

**Conclusion:**

Menu labeling effects were not uniform in this online fast-food ordering experiment. Their impact depends on consumers’ ability to interpret basic nutrition information. Designing more intuitive labels may improve the equity and effectiveness of restaurant nutrition policies.

## Introduction

Americans increasingly consume restaurant foods, which contribute substantially to excess calorie and sodium intake. In 2021-23, about one-third of US adults consumed fast food on a typical day, accounting for roughly 12% of total daily calories.^1^ Meals from fast-food restaurants are higher in calories, saturated fat, and sodium and lower in essential nutrients, such as calcium and fiber, compared to meals prepared at home.^2^ Poor diet remains a leading behavioral risk factor for mortality, responsible for nearly one-fifth of US deaths.^3^ Frequent fast-food consumption has been linked to obesity, cardiovascular disease, and type 2 diabetes.^4^ Importantly, these consumption patterns are shaped not only by individual choice but also by structural and environmental conditions, including the concentration of energy-dense, nutrient-poor options in many low-income communities (“food swamps”).^5^ Fast-food marketing is also disproportionately targeted toward socioeconomically disadvantaged populations and communities of color, potentially reinforcing existing dietary inequities.^6^

Menu labeling is a promising public health strategy aimed at improving consumer food choices in restaurants. The 2018 Affordable Care Act requirement to disclose calories on chain restaurant menus (ie, establishments with 20 or more locations) increased consumer awareness of nutrition information.^7^ However, evidence for its effectiveness is mixed, with observed reductions in calories ordered generally small and inconsistent across populations and settings.^8^ In contrast, sodium warning labels (including stoplight-style icons) have shown greater potential, with recent evaluations in the United States and United Kingdom indicating associations with decreased ordering of high-sodium menu items.^9^^,^^10^

Beyond federally mandated calorie disclosures on chain restaurant menus (and limited local requirements for nutrients such as sodium), restaurant menus often include unregulated descriptors such as “fresh,” “local,” “organic,” or “all natural.” These unregulated, sourcing-related claims are common but may not accurately identify items with healthier nutrient profiles.^11^ Prior research on packaged foods suggests such claims can shape perceptions of healthfulness, often creating “health halos” that mislead consumers to overestimate the healthfulness of products with claims.^12^^,^^13^ The effect of “health halos” may be especially pronounced among individuals with lower health literacy, who may be more susceptible to misleading or overly simplified product descriptors.

Health literacy, defined as the ability to obtain, understand, and apply health information, encompassing multiple domains, including functional, interactive, and critical health literacy,^14^ is a critical but underexamined factor that may influence how consumers respond to menu labeling and make food ordering decisions in restaurant settings. In the context of menu labeling, functional health literacy defined as the basic reading and numeracy skills needed to comprehend health information, is particularly relevant, as individuals must be able to interpret numeric nutrition information and visual cues to make healthier dietary choices.^15^^,^^16^ Yet, no prior research has examined how health literacy interacts with menu labeling to shape fast-food ordering behavior. This study addresses that gap by assessing whether consumers’ functional health literacy, measured using the Newest Vital Sign (NVS),^17^ moderates the effects of menu labels on calories and sodium ordered. Understanding this relationship can inform more accessible and effective nutrition labeling policies to improve population dietary health.

## Methods

### Participants

We conducted an anonymous online randomized controlled survey from April to May 2022 via CloudResearch Prime Panels (Prime Research Solutions LLC; Flushing, NY; 2015-22), accessing multiple research participant panel platforms in the United States. Recruitment used quota-based sampling to approximate US Census distribution for age and gender. CloudResearch’s built-in screening process, SENTRY, was used to detect duplicate, fraudulent, and inattentive respondents through digital fingerprinting, behavioral analysis, and evaluating response consistency across surveys so as to ensure high levels of response quality.^18^

**Figure 1:**
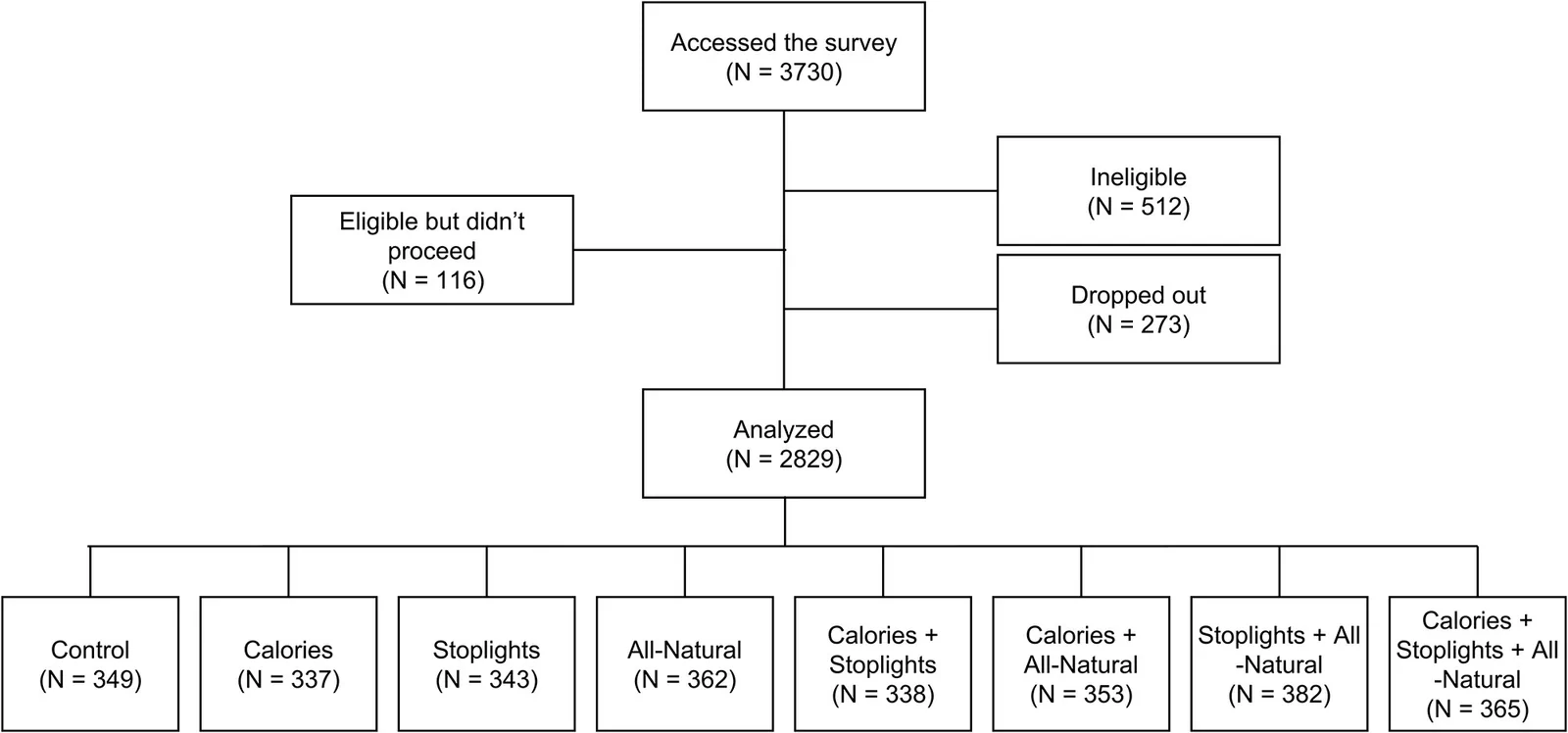
Participant flow diagram for survey recruitment, eligibility screening, and randomization to menu label condition.

A total of 3730 participants accessed the survey and were screened for eligibility. Of these, 2829 participants met inclusion criteria, completed the survey, and were included in the analytic sample (Figure 1). Participants were eligible if they were aged ≥18 years, resided in the United States, reported eating from a restaurant in the past month, were able to complete the survey in English, and provided informed consent. Participants received up to $6.00 in compensation through the panel platform. This study was approved by the host university’s Institutional Review Board.

Approximately half of the sample was from low-income households earning ≤$38 437 in 2021 dollars, the low-income threshold used in PEW’s online American Trends Panel.^19^ Participant demographic profiles from the panel platforms were used to purposefully recruit approximately half the sample from higher-income households and half the sample from lower-income households. Recruitment remained open for respondents until the prespecified quotas for each income group were reached. We wanted to explicitly compare low-income households with higher-income households, given the elevated risk for limited health literacy and diet-related chronic diseases low-income households experience.^20^ For analytic purposes, low-income status was subsequently defined using the 2022 US Department of Health and Human Services 185% Federal Poverty Level (FPL), accounting for household size.^21^

### Menu labels and food ordering

Participants were randomly assigned to 1 of 8 menu conditions in a 2 (calorie label: present vs. absent) × 2 (sodium stoplight label: present vs. absent) × 2 (all-natural label: present vs. absent) factorial design. Prices were displayed for each item. The calorie label condition displayed numeric calorie information next to each item. The sodium stoplight label condition used a red-yellow-green icon system to indicate warning (red), high (yellow), or low (green) sodium levels for each menu item.^10^ The all-natural label condition was designed to reflect common product sourcing claims frequently used by US restaurant chains,^11^ and included “ALL-NATURAL” for lemonade and “Natural-Cut with Sea Salt” for fries (Appendix Figure 1). Participants were then asked to select 1 entrée, 1 side, 1 beverage, and optionally 1 dessert from a drop-down list for each category, as if on a typical occasion. Total calories and sodium were selected as primary outcomes because they corresponded directly to the calorie and sodium label manipulations, are central targets of restaurant nutrition-labeling policies, and are highly relevant to diet-related chronic disease risk: excess calorie intake contributes to weight gain and obesity, and sodium is a key nutrient to limit in US dietary guidance and cardiovascular disease prevention.^22^^,^^23^

### Health literacy

We assessed health literacy using the NVS,^17^ because it directly assesses functional literacy and numeracy using nutrition-label information, making it well aligned with the skills required to interpret calorie and sodium information in a menu-labeling task. Its brief format also made it feasible for administration in an online survey. Participants viewed the nutrition label and responded to 6 questions (eg, “If you eat the entire container, how many calories will you eat?”). Correct answers were coded as 1, incorrect answers as 0, and total scores were summed to create the NVS total score, ranging from 0 to 6. Scores were categorized according to the established NVS scoring guidelines: A score of 0-1 suggests a high likelihood of limited literacy (“low literacy”); 2-3 indicates the possibility of limited literacy (“moderate literacy”); 4-6 indicates adequate literacy (“high literacy”). Among this sample, the NVS demonstrated good internal consistency with online administration and manual coding (Cronbach’s α = .75), comparable to in-person administration in the original validation study (Cronbach’s α = .76)^17^ and exceeding the benchmark value (α = .70), indicating reliable measurement with our online format and grading criteria.

### Sociodemographic factors

Sociodemographic information on gender, age, race, ethnicity, education level, income level, and current residential region was collected to describe sample characteristics (see Table 1 for response options). These sociodemographic characteristics were included as covariates in regression models to account for potential confounding in associations between menu labeling, health literacy, and ordering behaviors.

**Table 1:** Baseline demographic, fast food ordering behaviors, health literacy, and outcomes for total sample and by randomized group.

Participant characteristics	Total (*N* = 2829)	Control (*n* = 349)	Calories (*n* = 337)	Stoplights (*n* = 343)	All-Natural (*n* = 362)	Calories + Stoplights (*n* = 338)	Calories + All-Natural (*n* = 353)	Stoplights + All-Natural (*n* = 382)	Calories + Stoplights + All-Natural (*n* = 365)
**Demographics**									
** Age, M (SD)**	48.49 (17.48)	47.19 (16.77)	47.92 (18.03)	49.10 (17.14)	48.59 (17.12)	49.28 (16.98)	49.68 (17.72)	48.65 (18.17)	47.58 (17.83)
** Gender**									
** Female**	1466 (51.82%)	185 (53.01%)	163 (48.37%)	181 (52.77%)	201 (55.52%)	181 (53.55%)	191 (54.11%)	198 (51.83%)	166 (45.48%)
** Male**	1341 (47.40%)	163 (46.70%)	171 (50.74%)	162 (47.23%)	158 (43.65%)	155 (45.86%)	158 (44.76%)	180 (47.12%)	194 (53.15%)
**Non-binary/third gender**	14 (0.49%)	1 (0.29%)	3 (0.89%)	0 (0.00%)	1 (0.28%)	2 (0.59%)	3 (0.85%)	3 (0.79%)	3 (0.82%)
** Prefer not to say**	8 (0.28%)	0 (0.00%)	0 (0.00%)	0 (0.00%)	2 (0.55%)	0 (0.00%)	1 (0.28%)	1 (0.26%)	2 (0.55%)
** Hispanic or Latino**									
** No**	2500 (88.37%)	301 (86.25%)	304 (90.21%)	302 (88.05%)	318 (87.85%)	296 (87.57%)	318 (90.08%)	340 (89.01%)	321 (87.95%)
** Yes**	310 (10.96%)	46 (13.18%)	32 (9.50%)	39 (11.37%)	38 (10.50%)	41 (12.13%)	34 (9.63%)	40 (10.47%)	40 (10.96%)
** Prefer not to answer**	19 (0.67%)	2 (0.57%)	1 (0.30%)	2 (0.58%)	6 (1.66%)	1 (0.30%)	1 (0.28%)	2 (0.52%)	4 (1.10%)
** Race**									
** White/Caucasian**	2170 (76.71%)	254 (72.78%)	238 (70.62%)	265 (77.26%)	287 (79.28%)	268 (79.29%)	278 (78.75%)	294 (76.96%)	286 (78.36%)
**American Indian or Alaska Native**	32 (1.13%)	8 (2.29%)	4 (1.19%)	6 (1.75%)	2 (0.55%)	4 (1.18%)	3 (0.85%)	4 (1.05%)	1 (0.27%)
** Asian**	106 (3.75%)	11 (3.15%)	16 (4.75%)	9 (2.62%)	11 (3.04%)	12 (3.55%)	16 (4.53%)	14 (3.66%)	17 (4.66%)
**Black or African American**	307 (10.85)	43 (12.32%)	46 (13.65%)	38 (11.08%)	31 (8.56%)	34 (10.06%)	34 (9.63%)	48 (12.57%)	33 (9.04%)
** Multiracial**	121 (4.28%)	22 (6.30%)	19 (5.64%)	12 (3.50%)	17 (4.70%)	11 (3.25%)	11 (3.12%)	11 (2.88%)	18 (4.93%)
**Native Hawaiian or Other Pacific Islander**	11 (0.78%)	0 (0.00%)	3 (0.89%)	1 (0.29%)	1 (0.28%)	0 (0.00%)	2 (0.57%)	3 (0.79%)	1 (0.27%)
** Prefer not to answer**	22 (2.12%)	3 (0.86%)	2 (0.59%)	0 (0.00%)	5 (1.38%)	1 (0.30%)	5 (1.42%)	3 (0.79%)	3 (0.82%)
** Other**	60	8 (2.29%)	9 (2.67%)	12 (3.50%)	8 (2.21%)	8 (2.37%)	4 (1.13%)	5 (1.31%)	6 (1.64%)
** Education**									
** 8th grade or less**	13 (0.46%)	3 (0.86%)	0 (0.00%)	4 (1.17%)	1 (0.28%)	1 (0.30%)	1 (0.28%)	1 (0.26%)	2 (0.55%)
** Some high school**	114 (4.03%)	12 (3.44%)	13 (3.86%)	9 (2.62%)	16 (4.42%)	15 (4.44%)	15 (4.25%)	16 (4.19%)	18 (4.93%)
**High school graduate**	709 (25.06%)	101 (28.94%)	74 (21.96%)	88 (25.66%)	95 (26.24%)	92 (27.22%)	85 (24.08%)	92 (24.08%)	82 (22.47%)
**Job-specific training programs**	115 (4.07%)	12 (3.44%)	12 (3.56%)	19 (5.54%)	18 (4.97%)	13 (3.85%)	16 (4.53%)	10 (2.62%)	15 (4.11%)
**Some college but no degree**	628 (22.20%)	68 (19.48%)	74 (21.96%)	69 (20.12%)	86 (23.76%)	76 (22.49%)	80 (22.66%)	93 (24.35%)	82 (22.47%)
** Associate’s degree**	339 (11.98%)	45 (12.89%)	43 (12.76%)	42 (12.24%)	42 (11.60%)	42 (12.43%)	47 (13.31%)	41 (10.73%)	37 (10.14%)
** Bachelor’s degree**	576 (20.36%)	64 (18.34%)	74 (21.96%)	76 (22.16%)	59 (16.30%)	58 (17.16%)	67 (18.98%)	94 (24.61%)	84 (23.01%)
** Graduate degree**	335 (11.84%)	44 (12.61%)	47 (13.95%)	36 (10.50%)	45 (12.43%)	41 (12.13%)	42 (11.90%)	35 (9.16%)	45 (12.33%)
**Current residential region**									
** Midwest**	619 (21.88%)	91 (26.07%)	72 (21.36%)	75 (21.87%)	75 (20.72%)	70 (20.71%)	78 (22.10%)	83 (21.73%)	75 (20.55%)
** Northeast**	532 (18.81%)	58 (16.62%)	64 (18.99%)	66 (19.24%)	85 (23.48%)	62 (18.34%)	64 (18.13%)	67 (17.54%)	66 (18.08%)
** South**	1059 (37.43%)	130 (37.25%)	122 (36.20%)	140 (40.82%)	127 (35.08%)	128 (37.87%)	131 (37.11%)	149 (39.01%)	132 (36.16%)
** West**	619 (21.88%)	70 (20.06%)	79 (23.44%)	62 (18.08%)	75 (20.72%)	78 (23.08%)	80 (22.66%)	83 (21.73%)	92 (25.21%)
** Income (*N* = 2802)**									
** Above 185% FPL**	1694 (60.46%)	205 (58.74%)	212 (62.91%)	208 (60.64%)	217 (59.94%)	200 (59.17%)	193 (54.67%)	247 (64.66%)	212 (58.08%)
** Under 185% FPL**	1108 (39.54%)	138 (39.54%)	121 (35.91%)	134 (39.07%)	142 (39.23%)	136 (40.24%)	157 (44.48%)	133 (34.82%)	147 (40.27%)
**Fast-food consumption behaviors**									
**Fast-food consumption frequency**									
**Infrequent (0-2 times per week)**	1879 (66.42%)	228 (65.33%)	216 (64.09%)	222 (64.72%)	249 (68.78%)	236 (69.82%)	249 (70.54%)	251 (65.71%)	228 (62.47%)
**Frequent (3 or more times per week)**	950 (33.58%)	121 (34.67%)	121 (35.91%)	121 (35.28%)	113 (31.22%)	102 (30.18%)	104 (29.46%)	131 (34.29%)	137 (37.53%)
**Whether the hypothetical order matches with typical ordering behaviors in terms of quantity (*N* = 2826)**									
**Match typical order, with dessert**	1020 (36.09%)	122 (34.96%)	106 (31.45%)	126 (36.73%)	145 (40.06%)	118 (34.91%)	129 (36.54%)	143 (37.43%)	131 (35.89%)
**Match typical order, without dessert**	911 (32.24%)	111 (31.81%)	115 (34.12%)	110 (32.07%)	104 (28.73%)	122 (36.09%)	111 (31.44%)	125 (32.72%)	113 (30.96%)
**Match total item number but not under the same category**	106 (3.75%)	10 (2.87%)	17 (5.04%)	12 (3.50%)	14 (3.87%)	12 (3.55%)	14 (3.97%)	14 (3.66%)	13 (3.56%)
**Typically order fewer items**	162 (5.73%)	19 (5.44%)	25 (7.42%)	16 (4.66%)	22 (6.08%)	15 (4.44%)	17 (4.82%)	25 (6.54%)	23 (6.30%)
**Typically order more items**	627 (22.19%)	87 (24.93%)	74 (21.96%)	79 (23.03%)	77 (21.27%)	70 (20.71%)	80 (22.66%)	75 (19.63%)	85 (23.29%)
** Health literacy**									
** Low literacy**	860 (30.40%)	115 (32.95%)	112 (33.23%)	110 (32.07%)	102 (28.18%)	93 (27.51%)	92 (26.06%)	125 (32.72%)	111 (30.41%)
** Moderate literacy**	839 (29.66%)	101 (28.94%)	98 (29.08%)	99 (28.86%)	118 (32.60%)	106 (31.36%)	110 (31.16%)	102 (26.70%)	105 (28.77%)
** High literacy**	1130 (39.94%)	133 (38.11%)	127 (37.69%)	134 (39.07%)	142 (39.23%)	139 (41.12%)	151 (42.78%)	155 (40.58%)	149 (40.82%)
** Outcomes**									
**Total calories of the hypothetical order, *M* (SD)**	1620.91 (393.90)	1648.03 (389.75)	1617.25 (387.73)	1628.40 (418.74)	1627.64 (387.18)	1566.03 (397.99)	1613.85 (389.97)	1629.37 (379.08)	1633.39 (400.03)
**Total sodium of the hypothetical order, *M* (SD)**	1826.42 (481.42)	1860.93 (474.69)	1806.40 (502.30)	1850.64 (505.95)	1839.59 (458.70)	1767.40 (474.24)	1805.43 (494.22)	1834.05 (482.53)	1843.05 (456.90)

Denominators for percentages in each column are noted in the column headings. For variables with incomplete data, the total denominator is noted in the first column. Data reported in cells are the number in the category with percentages in parentheses unless otherwise noted in the first column for that row.

### Fast food consumption frequency

Participants reported the number of times in the past week they ate something from a fast-food restaurant alone or with other people. Response options ranged from 0 to 9 or more times. Based on their responses, participants were categorized into 2 groups: infrequent fast-food consumers (0-2 times per week) and frequent fast-food consumers (3 or more times per week).^24^

### Typical order comparison

After selecting 1 entrée, 1 side, 1 beverage, and optionally 1 dessert from the menu, participants were asked to indicate how many of each item (entree, side, beverage, dessert) they would typically order for themselves during a meal (from 0 to 10+ per category) to account for habitual ordering patterns.^25^ Because participants had the option of including dessert in their online order and could only select 1 of each food category (side, beverage, etc.), we also created several indicators based on how similar this experimental order was to their typical restaurant order. Experimental meal orders were categorized as: (1) Match typical order, without dessert (1 entree, 1 side, 1 beverage, no dessert); (2) match typical order, with dessert; (3) match total item number but different item categories (eg, 2 entrees, rather than an entree and a side); (4) typically order more items; and (5) typically order fewer items (see Appendix Table 1 for examples).

### Statistical analysis

All analyses were conducted in 2024 using RStudio (version 2023.06.2; RStudio Software, PBC, Boston, MA, USA). Chi-square tests and 1-way ANOVA were used to examine differences in sample characteristics across randomized groups. To test the primary research question, linear regression models were estimated separately for total calories and total sodium ordered. Unadjusted models examined bivariate associations between each predictor and the outcome. Adjusted models included interaction terms between menu label conditions and health literacy, controlling for sociodemographic characteristics, fast food consumption frequency, and whether participants’ hypothetical orders matched their typical ordering behavior. Diagnostic plots were examined to assess assumptions of linearity, normality, homoscedasticity, and independence of residuals. To aid interpretation of interaction terms, we estimated exploratory adjusted simple contrasts comparing each active labeling condition with the control condition within each health literacy group.

Because functional health literacy is associated with socioeconomic status and because income shapes food purchasing context, a sensitivity analysis was conducted by stratifying models by income level to assess whether the menu labeling × health literacy interaction was consistent across income groups. To assess whether cost influenced ordering patterns, we conducted a post hoc exploratory analysis examining whether total order cost according to the menu (Appendix Figure 1), calories per dollar, and sodium per dollar differed by income/SES indicators and whether including total order cost in adjusted calorie and sodium models altered the primary label × health literacy findings.

## Results

The 2829 participants included in the analysis were nationally representative in regard to age, gender, and race/ethnicity; about 40% had household incomes below 185% of the FPL. Of the 13 sample characteristics balance tests, none were statistically significant, indicating groups were well balanced across the 8 randomized experimental conditions. All sample characteristics are reported overall and by labeling condition in Table 1.

Regression results are shown in Table 2 with both unadjusted and adjusted estimates. The full model accounted for 38.4% of the variance in calories ordered (*R*² = 0.384, 95% CI: 0.35-0.40) and 12.2% of the variance in sodium ordered (*R*² = 0.122, 95% CI: 0.08-0.13). In unadjusted models, higher health literacy was associated with fewer calories and less sodium ordered; however, these associations were attenuated in adjusted models. Few menu-labeling conditions were significantly associated with calories or sodium ordered in the adjusted models, suggesting that labeling effects were not uniform across conditions.

**Table 2:** Multiple linear regression on the total calories and sodium from the hypothetical order.

	Calories				Sodium			
	Unadjusted model		Adjusted model		Unadjusted model		Adjusted model	
**Predictor**	*b*	*b* 95% CI [LL to UL]	*b*	*b* 95% CI [LL to UL]	*b*	*b* 95% CI [LL to UL]	*b*	*b* 95% CI [LL to UL]
**(Intercept)**			1862.47^a^	[1780.34 to 1944.59]			2063.85^a^	[1943.88 to 2183.82]
**Gender (Ref. = Female)**								
** Male**	127.42^a^	[98.61 to 156.24]	114.89^a^	[90.28 to 139.51]	119.04^a^	[83.63 to 154.44]	107.56^a^	[71.60 to 143.52]
** Non-binary/third gender**	−48.58	[−253.34 to 156.19]	−118.34	[−285.30 to 48.61]	−110.20	[−361.81 to 141.41]	−202.67	[−446.55 to 41.22]
** Prefer not to say**	72.27	[−198.06 to 342.61]	62.55	[−194.37 to 319.46]	−5.97	[−338.15 to 326.21]	91.94	[−283.36 to 467.24]
**Hispanic or Latino (Ref. = No)**								
** Yes**	136.18^a^	[89.93 to 182.43]	4.52	[−37.38 to 46.42]	99.32^a^	[42.57 to 156.06]	−6.26	[−67.46 to 54.95]
** Prefer not to answer**	49.62	[−127.27 to 226.50]	107.96	[−76.71 to 292.62]	−43.02	[−260.02 to 173.98]	−6.74	[−276.50 to 263.01]
**Race (Ref. White/Caucasian)**								
** American Indian or Alaska Native**	163.49^b^	[26.39 to 300.58]	102.24	[−11.28 to 215.76]	51.51	[−116.60 to 219.63]	4.70	[−161.13 to 170.53]
** Asian**	−36.21	[−112.79 to 40.37]	−92.07^a^	[−155.28 to −28.86]	−54.38	[−148.28 to 39.53]	−105.99^b^	[−198.33 to −13.65]
** Black or African American**	83.00^a^	[36.06 to 129.94]	−20.07	[−59.81 to 19.68]	12.48	[−45.09 to 70.04]	−73.83^b^	[−131.89 to −15.78]
** Multiracial**	76.20^b^	[4.28 to 148.11]	4.64	[−54.66 to 63.95]	77.56	[−10.62 to 165.75]	20.51	[−66.13 to 107.14]
** Native Hawaiian or Other Pacific Islander**	136.92	[−95.79 to 369.63]	59.38	[−127.88 to 246.63]	147.84	[−137.53 to 433.20]	91.75	[−181.79 to 365.29]
** Prefer not to answer**	−7.78	[−172.74 to 157.19]	−33.60	[−202.27 to 135.07]	−71.40	[−273.70 to 130.89]	−86.19	[−332.58 to 160.20]
** Some other race**	95.108	[−5.65 to 195.86]	43.61	[−43.54 to 130.75]	5.26	[−118.29 to 128.81]	−60.05	[−187.35 to 67.25]
**Age**	−5.99^a^	[−6.79 to −5.19]	−2.09^a^	[−2.88 to −1.29]	−5.24^a^	[−6.23 to −4.24]	−3.25^a^	[−4.41 to −2.08]
**Education (Ref. High school graduates)**								
** 8th grade or less**	97.39	[−118.04 to 312.83]	−1.40	[−173.44 to 170.65]	103.23	[−160.71 to 367.17]	28.40	[−222.91 to 279.72]
** Some high school**	6.65	[−71.03 to 84.32]	−4.06	[−67.18 to 59.06]	13.20	[−81.96 to 108.36]	−1.02	[−93.22 to 91.19]
** Job-specific training programs**	−108.19^a^	[−185.57 to −30.81]	−50.04	[−112.84 to 12.76]	−43.77	[−138.57 to 51.03]	−7.08	[−98.82 to 84.66]
** Some college but no degree**	−76.74^a^	[−118.92 to −34.56]	−41.45^b^	[−76.24 to −6.66]	−62.02^b^	[−113.70 to −10.35]	−42.50	[−93.33 to 8.32]
** Associate’s degree**	−78.63	[−129.4 to −27.80]	−46.07^b^	[−87.46 to −4.69]	−76.96^b^	[−139.23 to −14.69]	−52.18	[−112.63 to 8.28]
** Bachelor’s degree**	−57.34	[−100.52 to −14.17]	−47.33^b^	[−84.29 to −10.37]	−57.70^b^	[−110.60 to −4.80]	−54.77^b^	[−108.77 to −0.78]
** Graduate degree**	−88.94	[−139.97 to −37.90]	−96.55^a^	[−140.62 to −52.48]	−67.91^b^	[−130.44 to −5.39]	−69.52^b^	[−133.90 to −5.14]
**Current residential region (Ref. Midwest)**								
** Northeast**	6.62	[−38.93 to 52.16]	−21.27	[−58.15 to 15.61]	−17.56	[−73.34 to 38.22]	−37.07	[−90.95 to 16.81]
** South**	52.02^a^	[13.04 to 91.00]	16.48	[−15.36 to 48.33]	15.74	[−32.00 to 63.48]	−11.98	[−58.50 to 34.54]
** West**	79.52^a^	[35.73 to 123.31]	−0.69	[−37.07 to 35.69]	45.87	[−7.76 to 99.50]	−20.27	[−73.41 to 32.88]
**Health literacy level (Ref. Low literacy)**								
** Moderate literacy**	−117.70^a^	[−154.25 to −81.15]	101.02^b^	[15.88 to 186.16]	−55.46^b^	[−101.02 to −9.89]	130.16^b^	[5.79 to 254.53]
** High literacy**	−211.21^a^	[−245.29 to −177.13]	7.14	[−73.52 to 87.81]	−121.27^a^	[−163.76 to −78.78]	21.48	[−96.36 to 139.32]
**Fast food consumption frequency (Ref. Infrequent)**								
** Frequent**	170.91^a^	[140.81 to 201.01]	39.25^a^	[11.84 to 66.66]	124.47^a^	[87.16 to 161.77]	24.71	[−15.33 to 64.76]
**Income (Ref. Above 185% FPL)**								
** Under 185% FPL**	74.67^a^	[45.01 to 104.32]	17.35	[−9.10 to 43.81]	53.89^a^	[17.50 to 90.28]	18.70	[−19.95 to 57.34]
**Menu labels condition (Ref. Control)**								
** Calories**	−30.79	[−89.75 to 28.18]	43.53	[−38.90 to 125.96]	−54.53	[−126.59 to 17.53]	17.62	[−102.78 to 138.03]
** Stoplights**	−19.63	[−78.33 to 39.07]	5.39	[−77.16 to 87.95]	−10.29	[−82.03 to 61.44]	−11.66	[−132.25 to 108.94]
** All-Natural**	−20.40	[−78.32 to 37.52]	30.77	[−53.51 to 115.05]	−21.34	[−92.12 to 49.44]	32.12	[−90.99 to 155.23]
** Calories + Stoplights**	−82.00^a^	[−140.92 to −23.08]	−6.34	[−92.77 to 80.08]	−93.53^b^	[−165.54 to −21.53]	−96.43	[−222.69 to 29.82]
** Calories + All-Natural**	−34.18	[−92.47 to 24.10]	−16.13	[−103.59 to 71.32]	−55.50	[−126.72 to 15.72]	−65.64	[−193.39 to 62.12]
** Stoplights + All-Natural**	−18.66	[−75.83 to 38.51]	64.12	[−15.88 to 144.11]	−26.88	[−96.74 to 42.99]	65.87	[−50.98 to 182.73]
** Calories + Stoplights + All-Natural**	−14.64	[−72.44 to 43.16]	75.30	[−7.20 to 157.80]	−17.89	[−88.52 to 52.75]	66.93	[−53.59 to 187.45]
**Typical ordering behavior (Ref. Match typical order, with dessert)**								
** Match typical order, without dessert**	−466.06^a^	[−495.02 to −437.11]	−445.27^a^	[−474.10 to −416.43]	−269.31^a^	[−310.63 to −227.99]	−246.20^a^	[−288.32 to −204.08]
** Match total item number but not exactly the same order category**	−389.99^a^	[−454.81 to −325.17]	−417.23^a^	[−480.80 to −353.65]	−183.74^a^	[−276.24 to −91.23]	−203.74^a^	[−296.61 to −110.87]
** Typically order fewer items**	−311.58^a^	[−365.30 to −257.87]	−311.66^a^	[−364.31 to −259.00]	−132.81^a^	[−209.48 to −56.14]	−127.62^a^	[−204.54 to −50.70]
** Typically order more items**	30.43	[−1.81 to 62.66]	−28.73	[−63.40 to 5.94]	57.07^b^	[11.07 to 103.07]	10.17	[−40.48 to 60.81]
**Interaction between health literacy and menu labels (Ref. = Low literacy × Control)**								
** Moderate literacy × Calories**	−114.55	[−260.78 to 31.68]	−123.23^b^	[−243.73 to −2.73]	−147.50	[−329.78 to 34.78]	−161.59	[−337.61 to 14.43]
** High literacy × Calories**	−98.25	[−235.05 to 38.54]	−48.66	[−161.01 to 63.69]	−81.77	[−252.29 to 88.75]	−41.16	[−205.28 to 122.97]
** Moderate literacy × Stoplights**	−46.02	[−192.37 to 100.32]	−74.99	[−194.92 to 44.95]	10.25	[−172.17 to 192.67]	−18.56	[−193.76 to 156.65]
** High literacy × Stoplights**	−10.94	[−147.21 to 125.34]	−18.69	[−130.67 to 93.29]	14.59	[−155.29 to 184.46]	12.12	[−151.46 to 175.69]
** Moderate literacy × All-Natural**	−94.41	[−238.98 to 50.15]	−123.99^b^	[−242.89 to −5.10]	−135.31	[−315.51 to 44.90]	−155.60	[−329.27 to 18.08]
** High literacy × All-Natural**	−31.50	[−168.39 to 105.38]	−50.46	[−162.83 to 61.90]	−21.95	[−192.58 to 148.68]	−30.60	[−194.74 to 133.54]
** Moderate literacy × Calories + Stoplights**	−110.32	[−258.58 to 37.94]	−128.20^b^	[−249.99 to −6.42]	−37.82	[−222.63 to 146.99]	−41.11	[−219.01 to 136.79]
** High literacy × Calories + Stoplights**	−53.39	[−192.53 to 85.76]	−43.53	[−157.82 to 70.76]	38.41	[−135.04 to 211.86]	57.38	[−109.57 to 224.33]
** Moderate literacy × Calories + All-Natural**	−21.73	[−169.55 to 126.10]	−74.07	[−195.94 to 47.80]	−31.90	[−216.17 to 152.37]	−54.75	[−232.77 to 123.28]
** High literacy × Calories + All-Natural**	32.12	[−106.09 to 170.34]	18.96	[−95.09 to 133.01]	55.51	[−116.78 to 227.80]	67.74	[−98.87 to 234.34]
** Moderate literacy × Stoplights + All-Natural**	−167.99^b^	[−311.62 to −24.36]	−169.66^a^	[−287.73 to −51.59]	−171.36	[−350.40 to 7.68]	−178.84^b^	[−351.31 to −6.36]
** High literacy × Stoplights + All-Natural**	−73.76	[−205.60 to 58.07]	−68.44	[−176.52 to 39.64]	−100.41	[−264.75 to 63.92]	−91.32	[−249.20 to 66.56]
** Moderate literacy × Calories + Stoplights + All-Natural**	−219.14^a^	[−364.20 to −74.07]	−194.17^a^	[−313.38 to −74.96]	−228.59^b^	[−409.42 to −47.77]	−221.29^b^	[−395.43 to −47.15]
** High literacy × Calories + Stoplights + All-Natural**	−110.58	[−245.12 to 23.95]	−107.96	[−218.60 to 2.69]	−99.62	[−267.32 to 68.08]	−89.11	[−250.73 to 72.52]
**Model fit**			*R* ^2^ = .384^a^	95% CI [0.35 to 0.40]			*R* ^2^ = 0.122^a^	95% CI [0.08 to 0.13]

*b* represents unstandardized regression weights. LL and UL indicate the lower and upper limits of a confidence interval, respectively.

a *P* < .01.

b *P* < .05.

**Figure 2:**
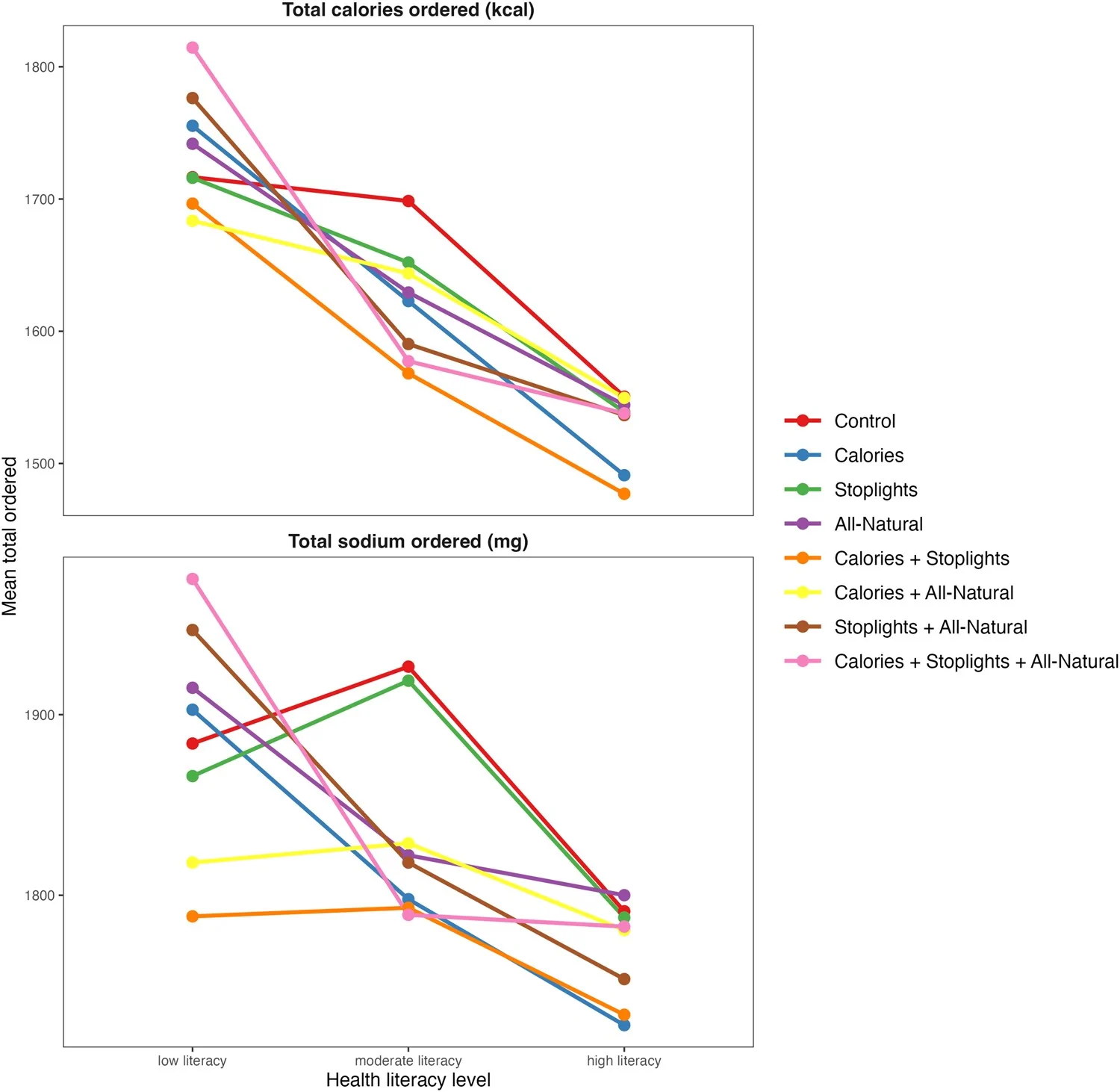
Mean calories and sodium ordered by menu label condition and health literacy level.

Several condition-specific treatment group × health literacy interaction terms were significant in the adjusted models (Table 2), indicating that label-versus-Control differences varied by health literacy for those conditions; descriptive group means are shown in Figure 2. However, the overall treatment group × health literacy interaction was not significant for calories, *F*(14, 2746) = 1.15, *P* = .305, or sodium, *F*(14, 2746) = 1.13, *P* = .325. Follow-up adjusted contrasts showed significant label-versus-Control differences only among participants with moderate health literacy. Among moderate-literacy participants, calorie selections were lower in the All-Natural, Calories + Stoplights, Calories + All-Natural, Stoplights + All-Natural, and Calories + Stoplights + All-Natural conditions than in Control (adjusted differences = −90.21 to −134.55 kcal, *P*s = .002-.037). Sodium selections were lower in the Calories, All-Natural, Calories + Stoplights, and Calories + Stoplights + All-Natural conditions than in Control (adjusted differences = −123.48 to −154.37 mg, *P*s = .016-.049). No significant label-versus-Control differences were observed among participants with low or high health literacy. The full set of adjusted contrasts is provided in Appendix Table 2.

Income-stratified sensitivity analyses for calories and sodium showed generally consistent patterns across income groups. However, several interaction terms between menu labels and moderate health literacy remained significant primarily among participants with lower income, particularly for the Calories + Stoplights, Stoplights + All-Natural, and Calories + Stoplights + All-Natural conditions, consistent with a potential 3-way interaction by income (Appendix Table 3).

Participants’ typical ordering behavior (Appendix Table 1) was a strong predictor of both calorie and sodium ordered. In this study, 68.33% of participants indicated that the fixed-item experimental order matched their typical fast-food restaurant order pattern. Compared to participants whose experimental choices mirrored their habitual orders (1 in each category: entree, side, beverage, and dessert), participants who normally ordered fewer items than what they selected in the experiment had orders that contained 311.66 fewer calories (95% CI: –364.31 to –259.00, *P* < .01) and 127.62 fewer milligrams of sodium (95% CI: –204.54 to –50.70, *P* < .01). Participants whose typical orders matched the number of items but not the categories (*b* = –417.23, 95% CI: –480.80 to –353.65, *P* < .01) or omitted dessert (*b* = –445.27, 95% CI: –474.10 to –416.43, *P* < .01) also ordered substantially less. In addition, frequent fast-food consumers selected orders with more calories than infrequent consumers (*b* = 39.25, 95% CI: 11.84-66.66, *P* < .01). Other significant predictors of less healthful consumption patterns included male gender, younger age, and lower educational attainment. Full coefficients and confidence intervals are provided in Table 2.

In post hoc exploratory analyses (Appendix Table 4), mean order cost was $12.40 (SD = $1.78). Participants below 185% FPL selected slightly higher-cost orders in unadjusted analyses (mean difference = $0.23, 95% CI: $0.10-$0.36), but this difference was not significant after adjustment (*b* = $0.03, 95% CI: −$0.07 to $0.14, *P* = .539). Adjusted differences in calories per dollar and sodium per dollar were also not significant, and mean cost did not differ across menu-labeling conditions (*F*(7, 2760) = 1.03, *P* = .409). Adding cost to the adjusted calorie and sodium models did not substantively alter the label × health literacy findings.

## Discussion

This randomized controlled trial tested the effectiveness of 3 menu labeling strategies (calorie labels, sodium stoplight labels, and all-natural labels), alone and in combination, on selections made in a hypothetical online fast-food ordering task. Among individuals with moderate health literacy, menu labels were associated with reductions in total calories and sodium ordered after accounting for sociodemographic factors, whereas these effects were not observed among participants with low or high health literacy. These findings have important implications regarding the real-world impact of implementing various menu labeling policies, which may have varying effects depending on consumers’ health literacy.

Among participants with moderate health literacy, multiple combinations of labels, including calorie labels alone, all-natural labels alone, and the combined calorie + sodium stoplight + all-natural labels, were associated with significantly lower calorie and sodium selected. Main effects of labeling and health literacy were not significant once covariates and interactions were accounted for. These patterns indicate that menu labeling is not uniformly effective but may exert the greatest influence when individuals possess sufficient functional health literacy to interpret the information. This finding suggests that menu labeling may be most effective among individuals with at least moderate health literacy who are not already making more healthful choices. Effects were not observed among high health literacy participants, possibly reflecting a ceiling effect; their baseline selections were already lower in calories and sodium, leaving limited room for improvement. This interaction effect between menu labels and health literacy may also help explain previously inconsistent findings in the labeling literature.^8^ Further, differences in health literacy measurement tools and domains assessed may also contribute to variability across studies,^26^ and aggregated analyses may mask meaningful differences across consumer segments.

In a national study assessing whether people noticed calorie labels on menus in 2017 and 2018,^27^ more than 70% of adults reported not using or noticing these labels, although the rates of label use seem to be rising since 2007.^28^ In the current study, one possible explanation is that individuals with higher health literacy selected healthier options regardless of the presence of menu labels in this individual food selection instance possibly because nutrition information has already been internalized and integrated into their habitual food selection process, whereas individuals with lower health literacy might have found these labels difficult to interpret or less noticeable. Labeling designs that are complementary to numeric labels (eg, more intuitive or visually salient designs) may help address these cognitive differences and warrant further study.^29^

Our demographic findings should be interpreted in the context of ordering behavior within an experimental fast-food menu-ordering task, rather than actual dietary intake quality. Consistent with prior work, older age, higher education, and female gender were associated with lower calories and sodium ordered.^30^^,^^31^ We also observed differences by race/ethnicity, with Asian participants ordering fewer calories and sodium than White participants, and Black participants ordering less sodium. While national survey data suggest racial/ethnic differences in overall diet quality,^32^^,^^33^ these findings are not directly comparable to the single-meal ordering outcomes assessed here. Instead, they may reflect differences in menu navigation, food preferences, or ordering norms within fast-food contexts.

Habitual ordering behavior was one of the strongest predictors of calories and sodium selection. Participants whose experimental choices mirrored their habitual orders (1 entree, 1 side, 1 beverage, and 1 dessert) selected the most calories and sodium, whereas those who omitted items, or varied category combinations placed orders that were substantially lower in calories and sodium. Even when the total item count was consistent, order composition (eg, substituting a side or beverage for a dessert) resulted in several hundred fewer calories, underscoring that what people habitually order may be as important as how much they order. Similarly, participants who typically ordered fewer items selected meals that were also less calorie- and sodium-dense when constrained by the experimental task. These findings align with research on habitual dietary patterns,^25^ which emphasizes that food choices are often automatic, stable, and resistant to change. Interventions that rely on conscious decision-making, such as menu labels, may therefore struggle to override entrenched habits. Future studies could explore integrating labeling with habit-disruption strategies (eg, changes in choice architecture, defaults, or reward systems).

The income-related findings further highlight equity considerations. Because food purchasing decisions are shaped by socioeconomic context, we explored whether labeling effects differed across income groups. Stratified sensitivity analysis indicated that labeling effects varied jointly by health literacy and income, with significant interactions observed primarily among lower-income participants. Given that functional health literacy varies by socioeconomic status, with lower-income groups more likely to fall within the moderate literacy range,^34^ labeling effects may be more detectable within this subgroup. Lower-income populations are disproportionately affected by diet-related chronic diseases^20^ and may benefit most from interventions that support healthier decision-making. These results suggest that well-designed labeling formats could contribute to reducing disparities in calories and sodium ordered within fast-food settings, although the underlying mechanisms remain uncertain. Exploratory cost analyses did not suggest that differences in meal cost explained the income-related patterns or the primary label × health literacy findings. However, future research should examine whether contextual factors such as food insecurity, pricing sensitivity, or targeted marketing practices amplify or constrain labeling effectiveness among lower-income groups using observed purchasing data.

### Public health implications

This study highlights the importance of considering individual differences in the design and implementation of nutrition-related public health interventions. Together, these findings suggest that the effectiveness of menu labeling depends in part on consumers’ capacity to interpret nutrition information. Tailoring menu labels to consumers with varying levels may require designs that are more visually salient and easier to interpret than the labels used in this study, such as larger icons, plain-language interpretive text, stronger visual contrast, or summary warnings that require less numerical interpretation.^35^ Fast food and quick-service restaurants remain critical intervention settings, as nearly 40% of adults report eating fast food on a given day.^36^ The ubiquity of fast food, combined with its disproportionate use among individuals with limited time or resources,^36^ underscores the need to evaluate labeling strategies in these environments. From a policy perspective, these findings support moving beyond “one-size-fits-all” labeling approaches and toward strategies that account for disparities in health literacy and other cognitive or socioeconomic factors. Public health efforts may also benefit from capacity-building initiatives that strengthen health literacy skills across functional, interactive, and critical domains. For example, community-based education programs or media literacy campaigns could help build more advanced health literacy skills to move individuals from low to moderate health literacy,^37^ potentially increasing the effectiveness of existing label formats. In practice, these efforts could include plain-language education through community health programs, clinics, workplace wellness programs, or community nutrition programs serving low-income adults. Using realistic fast-food menus, these programs could teach consumers how to compare calories, sodium icons, serving sizes, and marketing claims. Brief videos or infographics could accompany these education programs, ideally paired with more intuitive label designs that reduce cognitive burden at the point of purchase.

### Limitations

Several limitations should be considered. First, ordering behavior was assessed using a hypothetical online scenario rather than real-world purchasing data, which may not fully capture behavior in naturalistic settings. To partially address this issue, we assessed whether participants’ experimental selections matched their self-reported typical restaurant ordering patterns and adjusted for this variable in the primary models. Most participants (68.33%) indicated the task reflected their usual ordering patterns. However, this measure was also self-reported and cannot substitute for observed purchasing behavior. In real-world settings, choices may be influenced by price, time pressure, hunger, social context, sensory cues, promotions, and the use of personal funds. In addition, hypothetical ordering tasks may be vulnerable to social desirability or demand characteristics, which could lead participants to select healthier options than they would purchase in practice. Future studies should examine whether these labeling effects replicate in field experiments, digital ordering platforms, or restaurant transaction data.

Second, we did not assess participants’ health conditions (eg, obesity, hypertension, or cardiovascular disease), which could influence ordering decisions. Third, although our factorial design allowed for testing multiple label combinations, real-world food environments contain additional marketing or social cues that may alter the observed effects. Finally, the study focused on functional health literacy, which is directly relevant to interpreting menu labels, and did not assess other domains of health literacy such as interactive or critical literacy, which may further shape how individuals use nutrition information in real-world food environments.^38^

### Strengths

Despite these limitations, this study has several notable strengths. The use of a large, nationally representative sample enhances generalizability, and includes a substantial proportion of low-income participants at higher risks of diet-related diseases.^20^ The randomized controlled design allowed for causal inference regarding the effects of menu labeling conditions, and the fully crossed 2 × 2 × 2 factorial design enabled us to examine both independent and combined labeling strategies; while testing health literacy as an individual-level moderator. Adjustment for key sociodemographic covariates further strengthens the methodological rigor of the study.

## Conclusion

In this randomized online experiment, exposure to certain menu-labeling formats, particularly combinations of calorie, sodium stoplight, and all-natural labels, led to lower-calorie and lower-sodium selections in the online fast-food ordering task among individuals with moderate health literacy. Although menu labels showed no significant main effects overall, their impact emerged through interaction with consumer characteristics. These findings add important nuance to the literature by demonstrating that the effectiveness of menu labeling is contingent on consumers’ ability to interpret information. Identifying functional health literacy as a key moderator supports a more targeted, equity-oriented approach to nutrition labeling policy.

## Supplementary Material

kaag049_Supplementary_Data

## Data Availability

De-identified data from this study are not available in a public archive. De-identified data will be made available upon request to the corresponding author, in accordance with institutional IRB standards.
