# Supplementary material for: Health literacy as a moderator of menu labeling effects on fast-food ordering: an online randomized controlled trial
Source: Ann Behav Med. 2026 Aug 24;60(1):kaag049. doi: 10.1093/abm/kaag049 (PMC13502965; doi:10.1093/abm/kaag049)
Supplement: Supplementary file 1 [file kaag049_supplementary_data.zip › Appendix Table 4.docx]

**Appendix Table 4**

Post hoc exploratory cost analyses

*Panel A. Cost, calories-per-dollar, and sodium-per-dollar comparisons by income*

| *Outcome* | *Overall M (SD), N* | *Unadjusted below-minus-above 185% FPL difference [95% CI]* | *p* | *Adjusted B [95% CI]* | *p* |
| --- | --- | --- | --- | --- | --- |
| Total order cost, $ | 12.40 (1.78), 2829 | 0.23 [0.10, 0.36] | < .001 | 0.03 [-0.07, 0.14] | .539 |
| Calories per $ | 130.42 (23.72), 2829 | 3.42 [1.68, 5.16] | < .001 | 0.98 [-0.92, 2.87] | .312 |
| Sodium per $, mg | 147.27 (32.52), 2829 | 1.35 [-1.09, 3.79] | .277 | 0.90 [-1.83, 3.62] | .519 |

*Panel B. Primary model sensitivity after adding total order cost*

| *Outcome* | *Original label x literacy F(df1, df2)* | *p* | *Cost-adjusted label x literacy F(df1, df2)* | *p* | *Cost B [95% CI]* | *p* |
| --- | --- | --- | --- | --- | --- | --- |
| Total calories ordered | F(14, 2746) = 1.15 | .305 | F(14, 2745) = 1.20 | .265 | 108.69 [100.25, 117.12] | < .001 |
| Total sodium ordered | F(14, 2746) = 1.13 | .325 | F(14, 2745) = 1.03 | .420 | 186.98 [175.22, 198.73] | < .001 |

*Note.* Total order costs were calculated from displayed menu prices in dollars, using the mean displayed price for size-unspecified items. In Panel A, unadjusted differences are Welch mean differences; adjusted *B* values are HC3 heteroskedasticity-consistent model coefficients for participants below 185% FPL versus above 185% FPL. Panel A adjusted models controlled for gender, Hispanic/Latino ethnicity, race, age, education, region, health literacy, fast-food consumption frequency, menu-labeling condition, and typical-order match. Mean total order cost did not differ across menu-labeling conditions, *F(7, 2760) = 1.03, p = .409*. Panel B added total order cost to the adjusted calorie and sodium models that included menu-labeling condition × health literacy terms. CI = confidence interval.
