# Supplementary material for: Health literacy as a moderator of menu labeling effects on fast-food ordering: an online randomized controlled trial
Source: Ann Behav Med. 2026 Aug 24;60(1):kaag049. doi: 10.1093/abm/kaag049 (PMC13502965; doi:10.1093/abm/kaag049)
Supplement: Supplementary file 1 [file kaag049_supplementary_data.zip › Appendix Figure 1 Sample Menu Screens.docx]

**Appendix Figure 1. Sample menu screens from the Calories + Sodium Stoplight + All-Natural condition**

The sample condition displayed item prices, calorie information, sodium stoplight labels, and all-natural claims where applicable.

**Panel A. Entrées / Everyday Faves**

*Appendix Figure 1, continued*

**Panel B. Entrées / Made to Crave**

*Appendix Figure 1, continued*

**Panel C. Salads**

*Appendix Figure 1, continued*

**Panel D. Drinks and desserts**

*Appendix Figure 1, continued*

**Panel E. Kids’ Meal and Biggie Deals**

*Appendix Figure 1, continued*

**Panel F. Sides**
