# Supplementary material for: Health literacy as a moderator of menu labeling effects on fast-food ordering: an online randomized controlled trial
Source: Ann Behav Med. 2026 Aug 24;60(1):kaag049. doi: 10.1093/abm/kaag049 (PMC13502965; doi:10.1093/abm/kaag049)
Supplement: Supplementary file 1 [file kaag049_supplementary_data.zip › Appendix table 1.docx]

Appendix Table 1

Categorization of typical ordering behavior relative to experimental orders

| Ordering Category | Definition |
| --- | --- |
| Matched typical order, which included dessert | Participant reported typically ordering 1 entrée, 1 side, 1 beverage, and 1 dessert, and selected the optional dessert in the experiment. |
| Matched typical order, which included no dessert | Participant reported typically ordering 1 entrée, 1 side, 1 beverage, and no dessert, and did not select the optional dessert in the experiment. |
| Matched total items, different food item categories | Same number of items as experiment (e.g., four total) but distributed across different categories (e.g., 1 entrée, 2 sides, 1 beverage). |
| Typically order fewer items | Participant reported typically ordering fewer items than the 3–4 required in the experiment (e.g., only 1 entrée and 1 beverage). |
| Typically order more items | Participant reported typically ordering more than the 3–4 required in the experiment (e.g., 2 entrées, 1 side, 1 beverage, and 1 dessert). |

Notes: In the experiment, participants were required to select one entrée, one side, and one beverage, with the option to add a dessert. Thus, experimental orders always included 3 items (no dessert) or 4 items (with dessert).
