# Supplementary material for: Health literacy as a moderator of menu labeling effects on fast-food ordering: an online randomized controlled trial
Source: Ann Behav Med. 2026 Aug 24;60(1):kaag049. doi: 10.1093/abm/kaag049 (PMC13502965; doi:10.1093/abm/kaag049)
Supplement: Supplementary file 1 [file kaag049_supplementary_data.zip › Appendix Table 2 Adjusted Simple Contrasts.docx]

**Appendix Table 2**

*Exploratory adjusted simple contrasts comparing menu-labeling conditions with Control within health literacy groups*

| **Outcome** | **Health literacy group** | **Menu-labeling condition** | **Adjusted difference vs Control** | **95% CI** | *p* |
| --- | --- | --- | --- | --- | --- |
| Total calories ordered | Low | Calories | 43.53 | [-38.90, 125.96] | .301 |
| Total calories ordered | Low | Stoplights | 5.39 | [-77.16, 87.95] | .898 |
| Total calories ordered | Low | All-Natural | 30.77 | [-53.51, 115.05] | .474 |
| Total calories ordered | Low | Calories + Stoplights | -6.34 | [-92.77, 80.08] | .886 |
| Total calories ordered | Low | Calories + All-Natural | -16.13 | [-103.59, 71.32] | .718 |
| Total calories ordered | Low | Stoplights + All-Natural | 64.12 | [-15.88, 144.11] | .116 |
| Total calories ordered | Low | Calories + Stoplights + All-Natural | 75.30 | [-7.20, 157.80] | .074 |
| Total calories ordered | Moderate | Calories | -79.70 | [-167.69, 8.30] | .076 |
| Total calories ordered | Moderate | Stoplights | -69.59 | [-156.86, 17.67] | .118 |
| Total calories ordered | Moderate | All-Natural | -93.22 | [-177.21, -9.24] | .030 |
| Total calories ordered | Moderate | Calories + Stoplights | -134.55 | [-220.44, -48.65] | .002 |
| Total calories ordered | Moderate | Calories + All-Natural | -90.21 | [-175.11, -5.30] | .037 |
| Total calories ordered | Moderate | Stoplights + All-Natural | -105.54 | [-192.63, -18.46] | .018 |
| Total calories ordered | Moderate | Calories + Stoplights + All-Natural | -118.87 | [-205.07, -32.68] | .007 |
| Total calories ordered | High | Calories | -5.13 | [-81.43, 71.18] | .895 |
| Total calories ordered | High | Stoplights | -13.30 | [-88.68, 62.09] | .729 |
| Total calories ordered | High | All-Natural | -19.69 | [-93.96, 54.57] | .603 |
| Total calories ordered | High | Calories + Stoplights | -49.88 | [-124.62, 24.86] | .191 |
| Total calories ordered | High | Calories + All-Natural | 2.83 | [-70.45, 76.11] | .940 |
| Total calories ordered | High | Stoplights + All-Natural | -4.32 | [-77.06, 68.42] | .907 |
| Total calories ordered | High | Calories + Stoplights + All-Natural | -32.66 | [-106.52, 41.21] | .386 |
| Total sodium ordered | Low | Calories | 17.62 | [-102.78, 138.03] | .774 |
| Total sodium ordered | Low | Stoplights | -11.66 | [-132.25, 108.94] | .850 |
| Total sodium ordered | Low | All-Natural | 32.12 | [-90.99, 155.23] | .609 |
| Total sodium ordered | Low | Calories + Stoplights | -96.43 | [-222.69, 29.82] | .134 |
| Total sodium ordered | Low | Calories + All-Natural | -65.64 | [-193.39, 62.12] | .314 |
| Total sodium ordered | Low | Stoplights + All-Natural | 65.87 | [-50.98, 182.73] | .269 |
| Total sodium ordered | Low | Calories + Stoplights + All-Natural | 66.93 | [-53.59, 187.45] | .276 |
| Total sodium ordered | Moderate | Calories | -143.97 | [-272.51, -15.42] | .028 |
| Total sodium ordered | Moderate | Stoplights | -30.22 | [-157.69, 97.26] | .642 |
| Total sodium ordered | Moderate | All-Natural | -123.48 | [-246.16, -0.80] | .049 |
| Total sodium ordered | Moderate | Calories + Stoplights | -137.54 | [-263.01, -12.07] | .032 |
| Total sodium ordered | Moderate | Calories + All-Natural | -120.38 | [-244.40, 3.64] | .057 |
| Total sodium ordered | Moderate | Stoplights + All-Natural | -112.96 | [-240.18, 14.25] | .082 |
| Total sodium ordered | Moderate | Calories + Stoplights + All-Natural | -154.37 | [-280.28, -28.45] | .016 |
| Total sodium ordered | High | Calories | -23.53 | [-135.00, 87.93] | .679 |
| Total sodium ordered | High | Stoplights | 0.46 | [-109.66, 110.58] | .993 |
| Total sodium ordered | High | All-Natural | 1.52 | [-106.97, 110.01] | .978 |
| Total sodium ordered | High | Calories + Stoplights | -39.05 | [-148.23, 70.13] | .483 |
| Total sodium ordered | High | Calories + All-Natural | 2.10 | [-104.95, 109.14] | .969 |
| Total sodium ordered | High | Stoplights + All-Natural | -25.45 | [-131.70, 80.81] | .639 |
| Total sodium ordered | High | Calories + Stoplights + All-Natural | -22.18 | [-130.08, 85.72] | .687 |

*Note.* Values are adjusted simple contrasts estimated from the adjusted regression models. Each contrast compares the listed menu-labeling condition with the Control condition within the same health literacy group. Models adjusted for gender, Hispanic/Latino ethnicity, race, age, education, region, fast-food consumption frequency, income below 185% of the federal poverty level, and typical ordering behavior. Negative adjusted differences indicate fewer calories or less sodium selected compared with Control. CI = confidence interval.
