# Supplementary material for: Health literacy as a moderator of menu labeling effects on fast-food ordering: an online randomized controlled trial
Source: Ann Behav Med. 2026 Aug 24;60(1):kaag049. doi: 10.1093/abm/kaag049 (PMC13502965; doi:10.1093/abm/kaag049)
Supplement: Supplementary file 1 [file kaag049_supplementary_data.zip › Appendix Table 3 revised calories and sodium.docx]

**Appendix Table 3**

Income-stratified regression results for total calories and sodium ordered

|  | *Calories* | *Calories* | *Calories* | *Sodium* | *Sodium* | *Sodium* |
| --- | --- | --- | --- | --- | --- | --- |
| *Predictor* | *b (general)* | *b (low income)* | *b (high income)* | *b (general)* | *b (low income)* | *b (high income)* |
| (Intercept) | 1862.47** | 1883.19** | 1841.79** | 2063.85** | 2157.94** | 2002.86** |
| Gender (Ref. = Female) |  |  |  |  |  |  |
| Male | 114.89** | 116.52** | 110.01** | 107.56** | 108.34** | 102.44** |
| Non-binary / third gender | -118.34 | -157.26 | -2.19 | -202.67 | -339.79* | 71.46 |
| Prefer not to say | 62.55 | -67.17 | 224.66 | 91.94 | -27.38 | 156.84 |
| Hispanic or Latino (Ref. = No) |  |  |  |  |  |  |
| Yes | 4.52 | 12.34 | -2.18 | -6.26 | -46.29 | 31.26 |
| Prefer not to answer | 107.96 | 188.15 | 20.63 | -6.74 | 11.19 | -78.11 |
| Race (Ref. White/Caucasian) |  |  |  |  |  |  |
| American Indian or Alaska Native | 102.24 | 50.23 | 159.79 | 4.70 | 33.01 | -37.76 |
| Asian | -92.07** | -163.95** | -52.71 | -105.99* | -209.39* | -56.79 |
| Black or African American | -20.07 | -32.70 | 1.09 | -73.83* | -91.72* | -68.49 |
| Multiracial | 4.64 | -18.83 | 15.94 | 20.51 | -10.53 | 36.98 |
| Native Hawaiian or Other Pacific Islander | 59.38 | 0.31 | 103.48 | 91.75 | 55.16 | 127.21 |
| Prefer not to answer | -33.60 | -97.81 | 19.68 | -86.19 | -216.58 | 71.95 |
| Some other race | 43.61 | 31.03 | 59.61 | -60.05 | 0.83 | -130.79 |
| Age | -2.09** | -1.70** | -2.16** | -3.25** | -4.71** | -2.26** |
| Education (Ref. High school graduates) |  |  |  |  |  |  |
| 8th grade or less | -1.40 | -10.20 | -72.83 | 28.40 | 82.82 | -259.93 |
| Some high school | -4.06 | -12.23 | 26.17 | -1.02 | 1.62 | 22.94 |
| Job-specific training programs | -50.04 | -37.18 | -40.32 | -7.08 | 41.55 | -28.54 |
| Some college but no degree | -41.45* | -72.38** | -4.25 | -42.50 | -58.59 | -18.47 |
| Associate's degree | -46.07* | -56.13 | -23.63 | -52.18 | -11.74 | -64.84 |
| Bachelor's degree | -47.33* | -63.82 | -30.93 | -54.77* | -51.97 | -53.33 |
| Graduate degree | -96.55** | -79.24 | -89.87** | -69.52* | 65.31 | -87.71* |
| Current residential region (Ref. Midwest) |  |  |  |  |  |  |
| Northeast | -21.27 | -20.11 | -24.41 | -37.07 | -21.01 | -43.40 |
| South | 16.48 | 47.52 | -8.58 | -11.98 | -6.22 | -14.97 |
| West | -0.69 | 21.51 | -11.23 | -20.27 | -10.56 | -18.84 |
| Health literacy level (Ref. Low literacy) |  |  |  |  |  |  |
| Moderate literacy | 101.02* | 123.31 | 68.03 | 130.16* | 192.94* | 59.54 |
| High literacy | 7.14 | 5.89 | 6.03 | 21.48 | 26.34 | 15.27 |
| Fast food consumption frequency (Ref. Infrequent) |  |  |  |  |  |  |
| Frequent | 39.25** | 25.74 | 48.55* | 24.71 | 26.13 | 26.03 |
| Menu labels condition (Ref. Control) |  |  |  |  |  |  |
| Calories | 43.53 | 46.66 | 31.98 | 17.62 | -22.39 | 40.50 |
| Stoplights | 5.39 | -40.03 | 33.80 | -11.66 | -9.65 | 0.36 |
| All-Natural | 30.77 | 20.04 | 29.79 | 32.12 | 34.31 | 26.64 |
| Calories + Stoplights | -6.34 | -43.15 | 14.59 | -96.43 | -143.05 | -56.69 |
| Calories + All-Natural | -16.13 | -54.05 | 16.25 | -65.64 | -55.39 | -88.39 |
| Stoplights + All-Natural | 64.12 | 40.27 | 69.85 | 65.87 | 65.47 | 64.26 |
| Calories + Stoplights + All-Natural | 75.30 | 67.11 | 63.62 | 66.93 | 31.31 | 93.79 |
| Typical ordering behavior (Ref. Match typical order, with dessert) |  |  |  |  |  |  |
| Match typical order, without dessert | -445.27** | -456.39** | -435.07** | -246.20** | -251.95** | -238.37** |
| Match total item number but not exactly the same order category | -417.23** | -393.45** | -438.22** | -203.74** | -181.49* | -245.53** |
| Typically order fewer items | -311.66** | -240.59** | -340.51** | -127.62** | -54.93 | -160.83** |
| Typically order more items | -28.73 | -53.41* | -1.34 | 10.17 | -5.47 | 34.81 |
| Interaction between health literacy and menu labels (Ref. = Low literacy × Control) |  |  |  |  |  |  |
| Moderate literacy × Calories | -123.23* | -157.05 | -77.58 | -161.59 | -165.37 | -130.12 |
| High literacy × Calories | -48.66 | -76.61 | -25.26 | -41.16 | -113.43 | -18.89 |
| Moderate literacy × Stoplights | -74.99 | -19.02 | -122.91 | -18.56 | -57.75 | -8.91 |
| High literacy × Stoplights | -18.69 | 18.51 | -41.43 | 12.12 | 14.09 | -4.82 |
| Moderate literacy × All-Natural | -123.99* | -153.29 | -85.02 | -155.60 | -222.61 | -79.11 |
| High literacy × All-Natural | -50.46 | -20.10 | -52.92 | -30.60 | -37.03 | -17.57 |
| Moderate literacy × Calories + Stoplights | -128.20* | -200.43* | -51.58 | -41.11 | -146.77 | 51.25 |
| High literacy × Calories + Stoplights | -43.53 | -67.66 | -32.80 | 57.38 | 68.60 | 49.38 |
| Moderate literacy × Calories + All-Natural | -74.07 | -31.81 | -108.39 | -54.75 | -112.38 | 13.40 |
| High literacy × Calories + All-Natural | 18.96 | 25.34 | 7.19 | 67.74 | 42.41 | 91.15 |
| Moderate literacy × Stoplights + All-Natural | -169.66** | -208.75* | -114.55 | -178.84* | -295.88* | -75.90 |
| High literacy × Stoplights + All-Natural | -68.44 | -71.71 | -59.55 | -91.32 | -85.75 | -91.16 |
| Moderate literacy × Calories + Stoplights + All-Natural | -194.17** | -220.97* | -130.92 | -221.29* | -305.28* | -107.54 |
| High literacy × Calories + Stoplights + All-Natural | -107.96 | -97.92 | -95.51 | -89.11 | -44.88 | -118.11 |
| Model Fit | R² = .384** | R² = .373** | R² = .397** | R² = .122** | R² = .145** | R² = .121** |

*Note.* b represents unstandardized regression weights. Models are shown for the full sample and stratified by income group. Low income is defined as below 185% of the federal poverty level. * p < .05. ** p < .01.
